# A situation analysis of competences of research ethics committee members regarding review of research protocols with complex and emerging study designs in Uganda

**DOI:** 10.1186/s12910-021-00692-6

**Published:** 2021-09-26

**Authors:** Provia Ainembabazi, Barbara Castelnuovo, Stephen Okoboi, Walter Joseph Arinaitwe, Rosalind Parkes-Ratanshi, Pauline Byakika-Kibwika

**Affiliations:** 1 Infectious Diseases Institute, Makerere University, College of Health Sciences, P.O BOX 22418, Kampala, Uganda; 2 Department of Medicine, Makerere University College of Health Sciences, P.O Box 7072, Kampala, Uganda; 3 Clinical School, University of Cambridge, Cambridge, UK

**Keywords:** Complex and emerging study designs, Research Ethics Committees, Competence

## Abstract

**Background:**

Over the past two decades, Uganda has experienced a significant increase in clinical research driven by both academia and industry. This has been combined with a broader spectrum of research proposals, with respect to methodologies and types of intervention that need evaluation by Research Ethics Committees (RECs) with associated increased requirement for expertise. We assessed the competencies of REC members regarding review of research protocols with complex and emerging research study designs. The aim was to guide development of a training curriculum to improve the quality of scientific and ethical review.

**Methods:**

This was a cross-sectional study design, with quantitative data collection methods. Research Ethics Committee members completed a structured pre-coded questionnaire on current competence with complex and emerging study design. REC members were asked to outline a list of additional topics for which they needed training. Data from coded questions were entered into Epidata Version 3.1 and then exported to STATA Version14.1 for analysis. Descriptive analysis was performed and findings are presented using percentages and frequencies.

**Results:**

We enrolled 55 REC members from 6 RECs who have a total of 97 members. The majority of whom were males (56.4%, n = 31/55). The level of competence for review of selected study design was lowest for Controlled Human Infection Model (10.9%, n = 6) and reverse pharmacology design (10.9%, n = 6), and highest for cluster randomized study design (52.7%, n = 29) and implementation science research (52.7%, n = 29).

**Conclusion:**

Competence for review of research protocols with complex and emerging study design was low among participating REC members. We recommend prioritising training of REC members on complex and emerging study designs to enhance quality of research protocol review.

**Supplementary Information:**

The online version contains supplementary material available at 10.1186/s12910-021-00692-6.

## Background

Clinical research remains cardinal in advancing knowledge of disease, human biology, behavior and informs our health care practice [1]. In Uganda, there has been an up surge in clinical research driven by both academia and industry. Much of this focuses on the need to combat the emergence and re-emergence of infectious disease epidemics; including HIV, hemorrhagic fevers, tuberculosis, malaria, neglected tropical diseases, non-communicable diseases and the recent Severe Acute Respiratory Syndrome Corona Virus-2 that causes Corona virus disease-19 (COVID-19) [2–5]. This has broadened the spectrum of research activities in the quest for solutions to improve health and wellbeing.

Health research practice in Uganda has evolved over the years from mostly observation studies to more complex clinical interventional research. Documented early research using observational study designs focused on measuring incidence, prevalence and describing presentation of diseases. For example, Zika virus in 1947 and 1948, Burkitt’s lymphoma in the 1960s, Buruli ulcer in the early 1960s, Busoga hernia in 1964 and HIV/AIDs in the late 1980s [6–10].

With the coming of the HIV pandemic and introduction of antiretroviral therapy, scientists in Uganda embarked on conducting clinical trials with adherence and long-term efficacy monitoring [11, 12]. Recently, there has been increasing interest in vaccines research for infectious diseases and research for a cure for HIV [13] and more recently, COVID-19.

This research, which aimed to develop novel preventive, investigational and treatment strategies, is complex in design and involves significant ethical challenges—hence the need for competence to achieve high quality review and monitoring by Research Ethics Committees (REC) members, for knowledge generation while assuring for safety and wellbeing of humans as research participants.

Uganda’s research regulatory process mandates the RECs to review the science and ethics of research proposals, approve research protocols and oversee the conduct of clinical research with the aim of promoting high quality scientific research while minimizing risk to humans and ensuring respect for the research participant’s rights, values and interests [14].

Uganda has twenty-six [26] RECs accredited by the Uganda National Council for Science and Technology (UNCST). The REC accreditation is valid for three (3) years and is subject to continuing compliance with all applicable national standards and guidelines for RECs in Uganda, and to any additional stipulations or guidelines that may be provided by the UNCST [14].

RECs have mostly been reviewing observational studies since they are commonly conducted among studies approved at IDI Scientific Review committee between 2004 and 2017, observational studies make up 45%, clinical trials 19% and other types of research account for only 25%. Within clinical trials 59% are phase IV, 27% phase III and only 14% phase I/II (mostly vaccines) [15]. In a study conducted on academic research productivity of post-graduate students at Makerere University College of Health Sciences, Uganda, from 1996 to 2010, 75% of the post-graduate research projects were cross-sectional studies, with the least common designs being randomised trials (5%), diagnostic accuracy (3%) and economic evaluation (1%) [16]. However, there is increased conduct of clinical trials including phase I and II according to the National Drug Authority clinical trials website [16, 17].

Challenges arise when RECs are presented with protocols which stretch their expertise. The adaptation of complex and emerging research methods needs matched expertise by the REC members in order to conduct efficient and high quality scientific and ethical review.

It is therefore important to ensure that members of RECs have the competence to review research protocols to protect the safety, rights and welfare of research participants while advancing knowledge through high quality research. This study aimed to assess the competencies of REC members regarding review of research protocols with complex and emerging research study designs, in order to guide development of a relevant training curriculum for REC members.

## Methods

### Study design, site and population

This was a cross-sectional study among REC members from 6 UNCST accredited RECs located in Makerere University College of Health Sciences (MAKCHS) and Mulago National Referral Hospital, and Uganda Cancer Institute. The 6 RECs included; 4 RECs from each school of MAKCHS; School of Health Sciences REC (SHSREC), School of Medicine REC(SOMREC), School of Biomedical Sciences REC (SBSREC), School of Public Health Sciences REC (SPHREC), and the 2 were Mulago Hospital REC (MHREC) and Uganda Cancer Institute REC (UCIREC). The REC composition is guided by the UNSCT with the membership of 13–20 each. All REC members were eligible to enrol into the study if they were willing to participate.

### Study procedures

We planned to conduct face-face interviews with paper-based questionnaires, however, due to the lock-down restrictions following the COVID-19 pandemic, we changed to an online survey using the KoBoToolbx. The lists of REC members were obtained from the REC administrators with contact details: email and tele phone number. An email with a link to the online Informed Consent Form and the survey questionnaire were sent to all REC members inviting them to participate in the survey. In order to increase on the response rate, email and telephonic reminders were sent for participants who had not filled the survey after 7 and at 14 days of sending the questionnaire.

### Data collection

Data were collected using a pre-coded questionnaire in English language (Additional file 1). The questionnaire consisted of questions on demographic characteristics and self-reported competence in review of research protocols with complex and emerging study designs. The list of complex and emerging study design was generated from a consultative meeting with the REC chairpersons of the six RECs and reviewed by the study team and the study advisory board. Complex study design in this study referred to conventional research designs that apply methodologies that are not easy to design, analyse or understand. The complex study designs assessed included; adaptive, ecological, phase I, phase II, implementation science, step wedged design, and cluster randomized design. Emerging study design in this study referred to research designs that are increasingly being applied by researchers in the conduct of research in the low- and middle-income countries. The emerging study designs assessed included; controlled human infection model, reverse pharmacological design, and evaluation of studies on new technology/devices. The questions assessing the competence were on a Likert scale with four alternative responses with 1 = not competent, 2 = somewhat competent, 3 = competent, 4 = very competent. We used open-ended questions to collect data on other study designs that were not included on the list and asked for additional topics for which they needed training. The study coordinator checked the questionnaires for completeness.

### Data management and analysis

Data were entered into the study data base developed using Epi data version 3.1 released by Epi data Association, Odense, Denmark with in-built quality control checks. The final dataset was exported to STATA version 14.1 released by StataCorp for analysis. Competence of REC members was further categorized into two: competent and not competent and summarized using frequencies and percentages.

### Ethical consideration

The study was approved by School of Medicine REC (study reference number: #REC REF 2020-024), which is affiliated to Makerere University and accredited by UNCST (accreditation number: IRB00002062). The study also received approval from UNCST (study reference number: HS542ES). The REC chairpersons were informed about the survey before administering the questionnaire to REC members and Informed consent was obtained from each participant before administering the questionnaire.

## Results

### Social demographic characteristics of the participants

A total of 55 out of the 97 REC members contacted completed the survey questionnaire, giving a 56.7% response rate. All participants (23/23, 100%) that were contacted physically while 32 out of 74 participants contacted through email completed the survey. Of the 55 participants, 56.4% were male. Participants were from diverse background including; social sciences, bioethics, epidemiology and biostatistics, psychology, dentistry, education, medicine, oncology, public health, basic sciences, nursing and pharmacy as shown in Table 1 below. Majority of the participants had attained a Doctorate degree (PhD) (31/55, 56.4%). The highest number of participants from any REC was 14.

**Table 1:** Demographic characteristics of the participants

Variable	Frequency (percentage) (N = 55)
*Sex*	
Male	31 (56.4)
*Highest level of education*	
Certificate	01 (01.8)
Diploma	01 (01.8)
Bachelors	01 (01.9)
Masters	21 (38.2)
PhD	31 (56.4)
*REC*	
SHSREC	14 (25.5)
SOMREC	09 (16.4)
SBSREC	08 (14.6)
SPHREC	09 (16.4)
UCIREC	08 (14.6)
MHREC	07 (12.7)
*Background training*	
Medicine	11 (20.0)
Nursing	03 (5.5)
Social sciences	05 (09.1)
Bioethics	04 (07.3)
Basic sciences	06 (10.9)
Public health	15 (27.3)
Epidemiology and biostatistics	04 (07.3)
Dentistry	02 (03.6)
Psychology	01 (01.8)
Education	02 (03.6)
Pharmacy	02 (03.6)

### Competency regarding review of research protocols

**Fig. 1:**
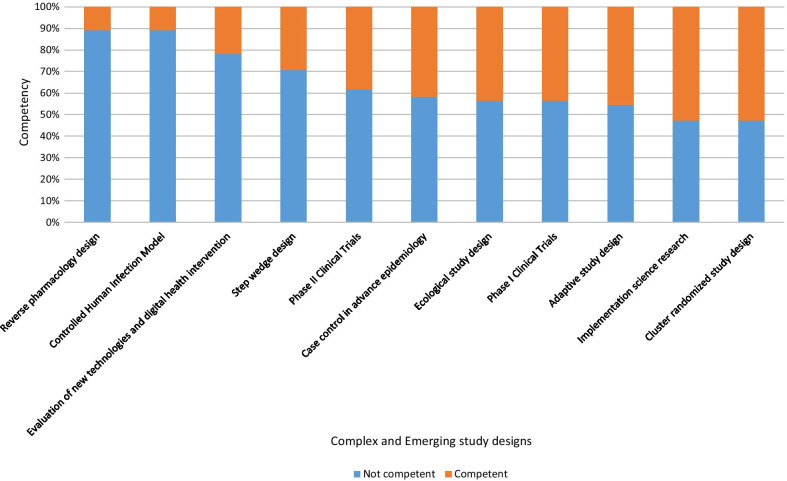
Proportion of REC members competent in review of complex and emerging study designs

The level of competence for review of selected complex and emerging study designs was lowest for controlled human infection model (10.9%, n = 6), reverse pharmacology design (10.9%, n = 6), and highest for cluster randomized study design (52.7%, n = 29), and implementation science research (52.7%, n = 29). The proportions of REC members competent to review other assessed study designs are; evaluation of new technology and digital health interventions (21.8%, n = 12), step wedged design (29.1%, n = 16), phase II clinical trials (38.2%, n = 21), case control with advanced epidemiology (41.8%, n = 23), ecological study design ( 43.6%, n = 24), phase I clinical trials (43.6%, n = 24), and adaptive study design (45.5%, n = 25) as shown in Fig. 1.

### Additional areas that pose challenges during review of research protocols

Some REC members reported difficulty reviewing research protocols for genetic studies; studies involving large longitudinal data, investigational new drug applications, new technology/devices, herbal medicine research as well as ethical dilemmas that arise with complex and emerging study designs.

## Discussion

We assessed the competencies of REC members regarding review of research protocols with complex and emerging research study designs to guide development of a relevant training curriculum to improve the quality of review. This study was initially designed to collect data using interviewer administered questionnaires, however, due to the COVID-19 pandemic and lockdown restrictions, we collected data using online self-administered questionnaires. Participants were from diverse background in line with the UNCST guidelines for composition of RECs in Uganda [14].

The response rate amongst REC members in this study was slightly higher compared to 52% in a similar study in a similar setting [18].

The majority of the participants reported lack of competence to review research protocols with complex and emerging study designs. The UNCST guidelines for composition of a REC require diversity of background of REC members. The guidelines also provide for an option to co-opt expertise to review research protocols when the REC members do not have adequate competence, and joint reviews may be arranged for review of complex and emerging study designs [14]. This remedy was also proposed by Bernard Lo and Deborah Grady as a way to strengthen REC review of highly innovative interventions in clinical trials [19]. Since REC members take full and collective responsibility to approve the research protocols, they need to be competent. The lack of competence reported could be due to the presence of members without an epidemiological background such as some social scientists and community representatives who are permanent members of the REC and without whom a REC review meeting cannot take place. Furthermore, there has been an increase in clinical interventional research driven by the changing disease patterns, emergence of new and highly infectious diseases plus the increased demand for research in emergency situations which presents more complex design and ethical challenges requiring refresher training.

Difficulty in review of complex study designs has been documented elsewhere. Lack of expertise was cited in a systematic review as one of the barriers hindering conduct of clinical trials in developing countries, lack of expertise was one of the cited barriers to their implementation in developing countries [20]. Chapman et al. highlighted that many RECs struggle with evaluating and providing oversight for innovative phase I trials because of lack of the technical competence to be able to conduct their own review [21].

Similar challenges were highlighted during the Global Forum on Bioethics in Research conferences [22] and the 10th Annual National Research Ethics Conference in Uganda [23]. The emergence of study designs like controlled human infection model, studies with digital intervention and genetic studies present enormous scientific and ethical challenges [24–26].

These challenges include establishing justification for deviating from the conventional trials, risk–benefit evaluation and informed consent. Stepped wedged trials and cluster randomised trial designs may need considerable thoughts on issues of scientific validity, practical, and logistical justification [27]. As Spencer Hey and colleagues noted, “new trial designs present challenges for assessing equipoise and discussing risks with patients and participants” [28]. For example, stepped wedged design delay the roll-out of the intervention to some of the control groups, the risks of delaying the roll-out should be considered [29]. In controlled human infection model studies, risk–benefit evaluation could be highly challenging since the interventions are tested on health volunteers that don’t stand to benefit clinically from the research [30].

The lack of competence in review of research protocols with complex and emerging study designs could lead to a longer or delayed research review process, poor quality review and rejection of important studies due to reluctance by the REC members to take on responsibility for novel trials being conducted in the country since all approved research projects must be monitored to completion. In addition, it may result in disinclination of many REC members to question the quality of the scientific data and their potential social value [31]. Instead, they may be inclined to defer to the judgment of one expert member rather than carefully scrutinizing the scientific rigour and ethical considerations for the protocols being presented.

### Limitations of the study

The small sample size which we attribute to the impact of COVID-19 restrictive measures affected our analysis and conclusions. All participants who were contacted in person completed the survey compared to 43% of those contacted through email filled the online survey. This indicates that response rate to the survey was greatly affected by the change in the mode of data collection because of COVID-19 restrictive prevention measures. This is consistent with previous studies that concluded that online surveys generally have a lower response rate compared to the in-person surveys [32, 33]. Few participants responded to the open-ended question and therefore this information could not be presented in the results as percentages. The results may not be generalisable to all RECs in the country due to the highly academic environment of the six RECs. There is limited literature on assessment of competence in assessed complex and emerging study designs amongst REC members in other settings. Thus, it is difficult to compare our findings to other places.

## Conclusion and recommendation

There is lack of competence in review of complex and emerging study design among the REC members studied and additional training in this area is an urgent priority. Results of this study have been used to guide development of a training curriculum for REC members in Uganda. The curriculum includes training on introduction to randomised controlled trials, phase I-IV clinical trials, adaptive study design, cluster randomized trial design, stepped wedged trial design, ecological study design, Implementation Science Research, controlled human infection model, reverse pharmacological design, and evaluation of studies with new technology/devices. Each session highlights key design issues, scientific and ethical issues that a REC member should pay attention.

## Supplementary Information

**Additional file 1.** A questionnaire for the assessment of competency of Research Ethics Committee members in review of research protocols with complex and emerging study designs.

## Data Availability

The datasets used and/or analysed during the current study are available from the corresponding author upon reasonable request. All data generated or analysed during this study are included in this published article.

## Abbreviations

REC: Research Ethics Committee
SOMREC: School of Medicine Research Ethics Committee
SHSREC: School of Health Sciences Research Ethics Committee
MHREC: Mulago Hospital Research Ethics Committee
UCIREC: Uganda Cancer Institute Research Ethics Committee
SBSREC: School of Biomedical Sciences Research Ethics Committee
SPHREC: School of Public Health Research Ethics Committee
COVID-19: Corona Virus Disease-19
