# Supplementary material for: A situation analysis of competences of research ethics committee members regarding review of research protocols with complex and emerging study designs in Uganda
Source: BMC Med Ethics. 2021 Sep 26;22:132. doi: 10.1186/s12910-021-00692-6 (PMC8474911; doi:10.1186/s12910-021-00692-6)
Supplement: Supplementary file 1 — Additional file 1. A questionnaire for the assessment of competency of Research Ethics Committee members in review of research protocols with complex and emerging study designs. [file 12910_2021_692_MOESM1_ESM.pdf]

SURVEY: NEEDS ASSESSMENT FOR NEW STUDY DESIGN CURRICULUM
QUESTIONNAIRE ID____

Background

Over the last two decades, Uganda has experienced a significant increase in clinical HIV that is driven by the changing HIV epidemic. This has broadened the spectrum of research activities that need to be evaluated by Research Ethics Committees (RECs) with associated requirement for new expertise. Reviewers over the years have been used to reviewing phase II, II and IV research and observation studies and challenges arise when they are presented with studies which stretch their expertise beyond this scope. Also, the scientific and ethical expertise uncertainties are usually compounded by current guideline gaps leaving researchers, research ethics committees and regulators with little support in how to evaluate, implement and run these study designs. This survey is therefore seeking for information on study designs where you find difficulty in reviewing both scientifically and ethically as a REC member as well as guideline gaps that in your experience could have hindered the research approval processes for the new study designs. The information gathered from this survey, will inform the design of a curriculum geared towards building capacity of REC members in reviewing complex study designs supported by the Ethics grant at Infectious Diseases Institute.

**SECTION 1: DEMOGRAPHIC CHARACTERISTICS**		
**#**	**Question**	**Response**
**1**	What is your sex?	1. Female<br>2. Male
**2**	Which institution are you affiliated to?	
**3**	What is your highest level of education?	
**4**	What is your area of expertise?	
**5**	Which REC(s) do you sit on?	
**6**	What is your role on the REC?	1. Scientific Review<br>2. Ethical Review<br>3. Both Scientific and Ethical Review
**SECTION 2: TRAINING NEEDS ON NEW STUDY DESIGNS**		
	On a scale of 1-4 with 1=Not competent, 2= somewhat competent, 3= competent, 4=very competent, how competent are you at reviewing research protocols with the following study designs.	
7	Adaptive study designs	1. Not competent<br>2. Somewhat competent<br>3. Competent<br>4. Very competent

MAKERERE UNIVERSITY
SCHOOL OF MEDICINE
APPROVED
VALID UNTIL
15 JAN 2021
RESEARCH & ETHICS COMMITTEE
P.O.BOX 7072, KAMPALA

**8**	Ecological studies	1. Not competent<br>2. Somewhat competent<br>3. 9Competent<br>4. Very competent
**9**	Phase I studies	1. Not competent<br>2. Somewhat competent<br>3. Competent<br>4. Very competent
**10**	Phase II studies	1. Not competent<br>2. Somewhat competent<br>3. Competent<br>4. Very competent
**11**	Implementation research	1. Not competent<br>2. Somewhat competent<br>3. Competent<br>4. Very competent
**12**	Cluster randomized design	1. Not competent<br>2. Somewhat competent<br>3. Competent<br>4. Very competent
**13**	Stepped wedged designs	1. Not competent<br>2. Somewhat competent<br>3. Competent<br>4. Very competent
**14**	Controlled human infection models	1. Not competent<br>2. Somewhat competent<br>3. Competent<br>4. Very competent
**15**	Reverse pharmacology design	1. Not competent<br>2. Somewhat competent<br>3. Competent<br>4. Very competent
**16**	Case-controls in advanced epidemiology	1. Not competent<br>2. Somewhat competent<br>3. Competent<br>4. Very competent

MAKERERE UNIVERSITY
SCHOOL OF MEDICINE
APPROVED
VALID UNTIL
15 JAN 2021
RESEARCH & ETHICS COMMITTEE
P.O.BOX 7072, KAMPALA

**17**	Evaluation of new technologies and digital health interventions	1. Not competent<br>2. Somewhat competent<br>3. Competent<br>4. Very competent
**18**	Any other study design that you have encountered as a REC member and think that REC members may need training on?	
**SECTION 3: GUIDELINE GAPS**		
**19**	What gaps in the existing UNCST and NDA guidelines have you identified as a REC member in review of complex study designs?	
**20**	Any other comments on complex study designs that you wish should be addressed?	
**Thank you for your response, we shall invite you to a dissemination meeting on the findings of this survey to plan the next steps.**		

MAKERERE UNIVERSITY
SCHOOL OF MEDICINE
APPROVED
VALID UNTIL
15 JAN 2021
RESEARCH & ETHICS COMMITTEE
P.O.BOX 7072, KAMPALA
